# Grief-related social media use and perceived social media support among bereaved students: associations with grief, mental health, and continuing bonds

**DOI:** 10.3389/fpubh.2026.1935184

**Published:** 2026-10-09

**Authors:** Caro Gerrits, Laurence Claes, Karl Andriessen

**Affiliations:** 1 Faculty of Psychology and Educational Sciences, KU Leuven, Leuven, Belgium; 2 Faculty of Medicine and Health Sciences, University Antwerp, Antwerp, Belgium; 3 Centre for Mental Health and Community Wellbeing, Melbourne School of Public Health, The University of Melbourne, Melbourne, VIC, Australia

**Keywords:** bereavement, grief, internet, interventions, mental health, social media, social support, students

## Abstract

**Background:**

Many students have experienced the loss of a close person during their academic career. This distressing event can have a strong impact on their daily and academic functioning and increases the risk of mental health problems. Social media provide a new space for grieving and maintaining continuing bonds with the deceased. While most students use social media applications daily, little is known of their grief-related social media use. The aim of this study was to examine the relationship of grief-related social media use and perceived social media support, with the grieving process, mental health, and continuing bonds among bereaved students.

**Methods:**

A total of 270 students, aged 18–28, from universities and institutions for higher education in Flanders/Belgium, filled out an online survey consisting of several questionnaires regarding grief, mental health and continuing bonds. Data were analyzed using correlation and hierarchical regression analyses.

**Results:**

Grief-related social media use was positively associated with stronger grief reactions and stronger continuing bonds with the deceased among bereaved students. Perceived social media support was associated with less severe grief reactions, but higher levels of mental distress. The findings indicate that bereaved students who engage more in grief-related social media activities may be at increased risk of experiencing grief and mental health problems.

**Conclusion:**

The study contributes to the limited body of research on grief-related social media use among students. Professional and peer support providers can play a crucial role by exploring the needs and coping strategies of bereaved students who engage with or seek support through social media and promote healthy coping and positive mental health outcomes.

## 1 Introduction

Approximately 40–70% of students experience the death of a loved one during their academic career (1) and about 18 to 30% have experienced such a loss over the past 12 months (2, 3). Grief can be described as the natural psychological, physical and behavioral response to loss (4). The death of a loved one can affect emerging adults and students in various ways, including emotionally, cognitively, somatically, and interpersonally (3, 5). For many of them, bereavement is a novel experience, and they may lack knowledge and life experience to deal with it. Experiencing the death of a close person can also cause students to become, to some extent, dependent on others again, such as parents (6). This can be confusing, as emerging adulthood is typically characterized by the pursuit of independence. Bereavement can therefore increase feelings of instability and the risk of psychosocial problems such as anxiety, depression, substance use (7, 8), and affect academic performance (9, 10).

Bereaved students may seek social support from family and friends (3, 11). Sharing their grief and feeling supported can alleviate feelings of isolation and the pain of the loss (12, 13). Experiencing social support may also contribute to personal growth, which in turn may positively affect mental health (14–16).

Bereaved students often engage in rituals to maintain a continuing bond with the deceased, such as talking about the deceased with others or visiting the deceased's social media profile (15, 17). A continuing bond is understood as the presence of an ongoing, internal relationship with a deceased person (18). Engaging in certain mourning rituals may help bereaved students to sustain this internal relationship. The literature indicates that maintaining continuing bonds can have both positive and negative effects on the grieving process (18, 19). Experiencing a burdensome continuing bond has been associated with higher levels of grief, maladaptive coping, and stigma in bereaved university students (17).

Approximately 95% of students aged 18 and older use social media applications on a daily basis (20). Due to their accessibility, these platforms have become spaces for bereaved students to express their grief, seek social support, and maintain continuing bonds with the deceased (21, 22). While websites for online memorials and other online grief resources have contributed to online grieving becoming more common (23, 24), research on grief-related social media use among students remains limited (1, 25).

Stein et al. (22) found that young adults (including students) post online messages to a deceased friend on important occasions, such as birthdays and holidays, and share recent events from their lives with the deceased. The study identified three functions of online memorial activities: (1) maintaining a continuing bond with the deceased, (2) giving and receiving information and social support, and (3) expressing their grief. Participants highly valued all three functions (22). Like the findings of Irwin (26), almost all participants in the study by Stein et al. (22) addressed their messages directly to the deceased, both in private and public posts. When participants addressed other Facebook users, they intended to share information, comfort others, or seek social support. More than half of the participants also regularly posted messages without a specific recipient, for example, when sharing photos or memories of the deceased (22).

A survey among adults identified three functions of grief-related Facebook use: (1) disseminating news about the death, (2) preserving memories of and the ongoing presence of the deceased, and (3) building a sense of community with others (27). Several of these functions were considered beneficial, including continued communication with the deceased and obtaining social support (27). Similarly, the study by Irwin (26) identified continued communication with the deceased as a beneficial function of grief-related Facebook use. Rossetto et al. (27) highlighted the importance of choice in maintaining a continuing bond with the deceased. Those who consciously choose to maintain an ongoing bond tend to view this positively. In contrast, those who do not consciously choose to maintain such a bond and are unexpectedly confronted with reminders of their loved one online are more likely to experience these encounters as distressing or hindering (27).

Findings from Lipp and O'Brien (1) further suggest that bereaved individuals who already experience symptoms of complicated grief may be more likely to turn to social media to connect with their deceased loved one. Social media use may then contribute to increased rumination, withdrawal from reality, or intense longing for the deceased (1).

Overall, the literature indicates that grief-related social media use may serve different functions, and the experience may vary depending on individual and contextual factors. Nonetheless, despite the devastating impact of bereavement on students and their daily usage of social media, limited research on grief-related social media use among students has been conducted. This study aimed to enhance our understanding of the relationship of grief-related social media use and perceived social media support, with the grieving process, mental health, and continuing bonds among bereaved students. More specifically, the study addressed the following three research questions.

Research Question 1: What is the association between grief-related social media use, perceived social media support, and the level of grief among bereaved students?

Research Question 2: What is the association between grief-related social media use, perceived social media support, and mental distress among bereaved students?

Research Question 3: What is the association between grief-related social media use, perceived social media support, and continuing bonds among bereaved students?

## 2 Materials and methods

### 2.1 Study design and sampling

We designed a cross-sectional study involving an online survey, created in Qualtrics (28). Eligible participants were (1) aged between 18 and 28 years, (2) enrolled in a Flemish college or university, (3) bereaved by the death of a close person after their 12th birthday (to minimize recall bias from childhood experiences), and (4) they had experienced the death at least six months before participation (to minimize the risk of recruiting participants in crisis shortly after bereavement). We have used these criteria successfully in previous studies [e.g., (5, 6)].

Participants could withdraw from the study at any time and received contact details of relevant support services. The study protocol received approval from the Social and Societal Ethics Committee of the KU Leuven (M-2023 01 019, January 2023).

An *a priori* sample size calculation using G^*^Power 3.1.9.7 (29) indicated that 138 participants would be needed for a two-tailed Spearman correlation analysis (α = 0.05, power = 0.95, *r*_*s*_ = 0.30). The sample size for a hierarchical multiple linear regression (α = 0.05, power = 0.95) with ten predictors and mean effect size (*f*^2^ = 0.15) was determined at 107.

The study announcement was widely disseminated through 403 student organizations, 20 Facebook groups, 14 grief organizations, and social media postings. Participants could enter a lottery to win one of five €20 gift vouchers. Between April and October 2022, a total of 557 individuals started the survey, 283 were excluded because of missing data or being ineligible. The survey included one question asking participants whether they had used social media after bereavement, and four who had not, were also excluded, resulting in a total sample of 270 participants (*M*_*age*_ = 21.83, *SD* = 2.14, female 78.9%, male 19.6%, other 1.5%). Table 1 presents the sociodemographic characteristics.

**Table 1:** Participant characteristics (*N* = 270).

Characteristic	*n*	%	
Gender			
Male	53	19.6	
Female	213	78.9	
Other	4	1.5	
Relationship with the deceased			
Parents	45	16.7	
Sibling	7	2.6	
Grandparent	165	61.1	
Other family member	18	6.7	
Friend or other	35	12.9	
Cause of death			
Natural death	205	75.9	
Accident	18	6.7	
Suicide	31	11.5	
Homocide	3	1.1	
Ohter/Unknown	13	4.8	
**Characteristic**	***M***	***SD***	**Min–max**
Age (years)	21.83	2.14	18–28
Emotional closeness to the deceased	7.9	1.8	1–10
Months since the death	44.81	33.46	6–170
Degree to which the death was expected	4.58	3.61	0–10
CBS	0.93	0.52	0–2.31
AGI	2.26	0.58	1.03–3.97
CBSMS	20.99	8.53	14–57
FMSS	31.57	6.91	14–51
DASS−21	18.37	13.92	0–63

CBS, Continuing Bonds Scale (30); AGI, Adolescent Grief Inventory (31); CBSMS, Continuing Bonds on Social Media Scale (1); FMSS, Facebook Measure of Social Support (32); DASS−21, Depression Anxiety Stress Scale 21 (33).

### 2.2 Survey

**Sociodemographic questions**: Participants received sociodemographic questions regarding their age and sex, and their relationship to the deceased person, including type of relationship, emotional closeness, time since death (years and months), cause of death (natural, accident, suicide, homicide, other/unknown), and expectedness of death (Table 1). Emotional closeness and expectedness of death were rated on a scale from 0 to 10, with higher scores indicating stronger closeness or expectedness. We adopted these questions from previous studies (5, 11).

The **Continuing Bonds Scale** [CBS, (30)] consists of 16 items measuring internalized (e.g., “I thought about the deceased as a role model who I try to be like”), and externalized continuing bonds (e.g., “I actually heard the voice of the deceased speak to me”). Participants rated each item on a 4-point Likert-type scale, ranging from “0 = Never applicable” to “3 = Usually applicable,” indicating to what extent it applied to them over the last month. The mean scores were calculated (range 0–3), with higher scores indicating higher levels of continuing bonds. The Cronbach's alpha coefficient in this sample was 0.89.

The **Adolescent Grief Inventory** [Dutch version AGI, (5, 16, 31)] consists of 40 items and measures the level of grief (e.g., “I worried that other people would die as well”) across six domains, i.e., sadness, self-blame, anxiety and self-harm, shock, anger and betrayal, sense of peace. Participants rated each item on a 5-point Likert-type scale, ranging from “1 = Not at all” to “5 = Extremely,” indicating how much they were applicable over the past month. We calculated mean scores (range 1–5), with higher scores indicating stronger grief reactions. The Cronbach's alpha coefficient was 0.91.

The **Continuing Bonds on Social Media Scale** [CBSMS, (1)] measures maintaining an ongoing bond with the deceased using social media. The 14 items address seven themes, i.e., general activity, memories, updates, appreciation, ritual, presence, and eventual reunion. Each item is rated on a 5-point scale, ranging from “1 = Never” to “5 = Very often,” indicating the extent of participants' engagement on social media after bereavement. Item scores were summed (range 14–70), with higher scores indicating higher levels of using social media to sustain continuing bonds. The Cronbach's alpha coefficient was 0.90.

The **Facebook Measure of Social Support** [FMSS, (32)] was originally developed to measure the perceived social support obtained through Facebook. Similar to Lipp and O'Brien (1), we replaced “Facebook” with “social media” to better align the 14 items with our study (e.g., “The support I get on *social media* makes me feel better”). Participants rated each item on a 5-point scale, ranging from “1 = Never” to “5 = Almost always,” indicating how much it applied to them since bereavement. The first item was reversed scored, and item scores were summed (range 14–70), with higher scores indicating higher perceived social support. The Cronbach's alpha coefficient was 0.76.

The **Depression Anxiety Stress Scale-21** [Dutch version DASS-21, (33, 34)] was used to measure mental distress. Participants rated the extent to which each of the 21 items (e.g., “I found it hard to wind down”) applied to them over the last week on a 4-point Likert-type scale, ranging from “0 = Never” to “to ‘3 = Almost always”. Item scores were summed (range 0–63) and higher scores indicated higher levels of mental distress. The Cronbach's alpha coefficient was 0.96.

### 2.3 Translation of measures

We translated the Continuing Bonds Scale [CBS, (30)], the Continuing Bonds on Social Media Scale [CBSMS, (1)], and the Facebook Measure of Social Support [FMSS, (32)] from English into Dutch following the recommended procedure (35). This included: (1) forward translation, (2) expert panel review, (3) back translation, (4) review of disagreements, and (5) pre-testing of the translated version.

### 2.4 Data analysis

Data were analyzed using SPSS 29 (36). We used descriptive statistics to analyse the sociodemographic data (Table 1), and Spearman's correlation analysis to investigate the relationships between the study variables (Table 2).

**Table 2:** Spearman correlations (*N* = 270).

Variable	1	2	3	4	5	6	7	8	9	10	11	12
1. Gender	–											
2. Age	-0.102	–										
3. Type of relation	-0.080	−0.018	–									
4. Emotional closeness to the deceased	0.098	0.052	-0.182^**^	–								
5. Months since the death	-0.030	**0.210** ^**^	-0.159^**^	0.110	–							
6. Cause of death	-0.061	−0.028	**0.211** ^**^	−0.100	-0.091	–						
7. Expectedness of death	-0.065	0.022	-0.046	−0.050	0.003	-0.339^**^	–					
8. CBS	**0.191** ^**^	−0.112	-0.121^*^	**0.533** ^**^	-0.130^*^	−0.035	-0.097	–				
9. AGI	**0.149** ^*^	-0.146^*^	0.004	**0.210** ^**^	-0.211^**^	**0.212** ^**^	-0.233^**^	**0.470** ^**^	–			
10. CBSMS	**0.187** ^**^	−0.077	0.073	**0.229** ^**^	-0.036	**0.179** ^**^	-0.257^**^	**0.390** ^**^	**0.406** ^**^	–		
11. FMSS	**0.182** ^**^	−0.036	0.063	−0.059	-0.058	0.008	0.019	0.007	0.017	**0.260** ^**^	–	
12. DASS−21	**0.135** ^*^	-0.148^*^	0.100	0.088	-0.098	**0.143** ^*^	-0.119	**0.345** ^**^	**0.590** ^**^	**0.230** ^**^	**0.151** ^*^	–

^*^p < 0.05, ^**^p < 0.01.

CBS, Continuing Bonds Scale (30); AGI, Adolescent Grief Inventory (31); CBSMS, Continuing Bonds on Social Media Scale (1); FMSS, Facebook Measure of Social Support (32); DASS-21, Depression Anxiety Stress Scale 21 (33).

To examine the three research questions, we performed three hierarchical regression analyses. The assumptions underlying the hierarchical regression analyses (e.g., multicollinearity, normality of residuals, homoscedasticity, and linearity) were evaluated before conducting the analyses. Multicollinearity was assessed using tolerance and variance inflation factor (VIF) values, with tolerance values >0.10 and VIF values <5 considered acceptable. Normality of the residuals was examined using a histogram and a normal P–P plot of the standardized residuals. Linearity and homoscedasticity were evaluated by visually inspecting the scatterplot of standardized residuals against standardized predicted values. The absence of systematic or curved patterns and a relatively constant spread of residuals across predicted values indicated that these assumptions were adequately met. For each of these analyses, age, sex, and variables that significantly correlated with the dependent variable and at least one independent variable were included as control variables in Step 1. In Step 2, the use of social media to sustain continuing bonds (CBSMS; 1), and the perceived social support received through social media (FMSS; 32), were included as potential predictors of each of the dependent variables, i.e., grief (AGI; 31), mental distress (DASS-21; 33), or continuing bonds [CBS, (30)]. While we used the word “predictor” to describe findings of the regression analyses, we acknowledge that the study involved cross-sectional data.

## 3 Results

### 3.1 The relationships between the study variables

The correlation analysis (Table 2) found a significant positive correlation between grief-related social media use (CBSMS) and continuing bonds (*r*_*s*_ = 0.390, *p* < 0.01), grief reactions (*r*_*s*_ = 0.406, *p* < 0.01), sex (*r*_*s*_ = 0.187, *p* < 0.01), emotional closeness (*r*_*s*_ = 0.229, *p* < 0.01) and cause of death (*r*_*s*_ = 0.179, *p* < 0.01), and a negative correlation between grief-related social media use and expectedness of death (*r*_*s*_ = −0.257, *p* < 0.01).

The perceived social media support (FMSS) showed a positive correlation with sex (*r*_*s*_ = 0.182, *p* < 0.01) and the grief-related social media use (*r*_*s*_ = 0.260, *p* < 0.01).

The level of grief (AGI) had a positive correlation with continuing bonds (*r*_*s*_ = 0.470, *p* < 0.01), sex (*r*_*s*_ = 0.149, *p* < 0.05), emotional closeness (*r*_*s*_ = 0.210, *p* < 0.01) and cause of death (*r*_*s*_ = 0.212, *p* < 0.01), and a negative correlation with age (*r*_*s*_ = −0.146, *p* < 0.05), time since death (*r*_*s*_ = −0.211, *p* < 0.01) and expectedness of death (*r*_*s*_ = −0.233, *p* < 0.01).

Mental distress (DASS-21) correlated positively with grief reactions (*r*_*s*_ = 0.590, *p* < 0.01), continuing bonds (*r*_*s*_ = 0.345, *p* < 0.01), sex (*r*_*s*_ =0.135, *p* < 0.05), cause of death (*r*_*s*_ =0.143, *p* < 0.05), grief-related social media use (*r*_*s*_ = 0.230, *p* < 0.01) and the perceived social media support (*r*_*s*_ = 0.151, *p* < 0.05). Mental distress also showed a negative correlation with age (*r*_*s*_ = −0.148, *p* < 0.05).

There was a positive correlation between continuing bonds (CBS) and sex (*r*_*s*_= 0.191, *p* < 0.01) and emotional closeness (*r*_*s*_ = 0.533, *p* < 0.01), and a negative correlation between continuing bonds (CBS) and type of relationship (*r*_*s*_ = −0.121, *p* < 0.05) and number of months since death (*r*_*s*_ = −0.130, *p* < 0.05).

### 3.2 The association between grief-related social media use, perceived social media support, and the level of grief

All relevant assumptions, including linearity, normality and homoscedasticity of residuals, and the absence of problematic multicollinearity, were adequately met, supporting the use of hierarchical linear regression analyses.

Age, sex, emotional closeness, time since death, cause of death, expectedness of death, continuing bonds (CBS) and mental health (DASS-21) were included in Step 1 [*F* (8,261) = 28.136, *p* < 0.001, *R*^2^ = 0.463]. In Step 2, the use of social media to sustain continuing bonds (CBSMS), and the perceived social support received through social media (FMSS), were included as potential predictors of level of grief as dependent variables (Table 3). The regression model including step 2 with the level of grief (AGI) as dependent variable was statistically significant and explained 51% of the variance [*F* (10,259) = 26.966, *p* < 0.001, *R*^2^ = 0.510; ΔR^2^ = 0.047, *F* (2, 259) = 12.431, *p* < 0.001]. The use of social media to sustain continuing bonds (CBSMS) (β = 0.246, *p* < 0.001), and the perceived social support received through social media (FMSS) (β = −0.127, *p* < 0.05) were significant predictors of the level of grief (AGI). More social media usage was associated with higher levels of grief, and higher levels of perceived social support were associated with lower levels of grief.

**Table 3:** Hierarchical regression analysis predicting the grief process (AGI) from grief-related social media use and perceived social support on social media (*N* = 270).

Model		Unstandardized coefficients		Standardized coefficients beta	*t*	*p*
		B	Std. Error			
1	(Constant)	1.661	0.335		4.954	<0.001
	Gender	0.061	0.064	0.044	0.947	0.344
	Age	−0.005	0.013	−0.018	-0.378	0.706
	Emotional closeness to the deceased	0.020	0.018	0.061	1.072	0.285
	Months since death	−0.002	0.001	−0.129	-2.681	0.008
	Cause of death	0.046	0.027	0.084	1.735	0.084
	Expectedness of death	−0.023	0.008	−0.143	-2.997	0.003
	CBS	0.238	0.067	0.213	3.558	<0.001
	DASS−21	0.010	0.001	0.458	9.152	<0.001
	*R^2^*	0.463				
	Adjusted *R^2^*	0.447				
2	(Constant)	1.810	0.336		5.380	<0.001
	Gender	0.058	0.063	0.042	0.919	0.359
	Age	−0.005	0.013	−0.018	-0.387	0.699
	Emotional closeness to the deceased	0.008	0.018	0.025	0.452	0.652
	Months since death	−0.002	0.001	−0.137	-2.981	0.003
	Cause of death	0.035	0.026	0.064	1.387	0.167
	Expectedness of death	−0.016	0.008	−0.097	-2.082	0.038
	CBS	0.169	0.066	0.151	2.576	0.011
	DASS−21	0.009	0.001	0.437	8.954	<0.001
	CBSMS	0.017	0.004	0.246	4.775	<0.001
	FMSS	−0.011	0.004	−0.127	−2.704	0.007
	*R^2^*	0.510				
	Adjusted *R^2^*	0.491				
	ΔR^2^ (*p* < 0.001)	0.047				

CBS, Continuing Bonds Scale (30); AGI, Adolescent Grief Inventory (31); CBSMS, Continuing Bonds on Social Media Scale (1); FMSS, Facebook Measure of Social Support (32); DASS-21, Depression Anxiety Stress Scale 21 (33).

### 3.3 The association between grief-related social media use, perceived social media support, and mental distress

Age, sex, cause of death, continuing bonds (CBS), and level of grief (AGI) were included in Step 1 [*F* (5,264) = 29.695, *p* < 0.001, *R*^2^ = 0.360]. In Step 2, the use of social media to sustain continuing bonds (CBSMS), and the perceived social support received through social media (FMSS), were included as potential predictors of mental distress (DASS-21) (Table 4). The regression model including step 2 with mental distress (DASS-21) as dependent variable was statistically significant and explained 37.8% of the variance [*F* (7,262) = 22.792, *p* < 0.001, *R*^2^ = 0.378; ΔR^2^ = 0.019, *F* (2, 262) = 3.901, *p* < 0.021]. The perceived support from social media (FMSS) (β = 0.144, *p* < 0.05) was a significant predictor of mental distress (DASS-21). Higher levels of perceived social support through social media were associated with lower levels of mental distress. The use of social media to sustain continuing bonds (CBSMS) (β = −0.037, *p* > 0.05) was not a significant predictor of mental distress (DASS-21).

**Table 4:** Hierarchical regression analysis predicting mental distress (DASS-21) from grief-related social media use and perceived social support on social media (*N* = 270).

Model		Unstandardized coefficients		Standardized coefficients beta	*t*	*p*
		B	Std. Error			
1	(Constant)	−11.953	17.180		−0.696	0.487
	Gender	1.613	3.338	0.025	0.483	0.629
	Age	−0.786	0.650	−0.060	−1.208	0.228
	Cause of death	1.043	1.330	0.040	0.784	0.434
	CBS	5.730	2.976	0.107	1.926	0.055
	AGI	24.841	2.707	0.520	9.175	<0.001
	*R^2^*	0.360				
	Adjusted *R^2^*	0.348				
2	(Constant)	−28.007	17.951		−1.560	0.120
	Gender	0.006	3.369	0.000	0.002	0.999
	Age	−0.733	0.645	−0.056	−1.137	0.256
	Cause of death	1.057	1.319	0.040	0.801	0.424
	CBS	6.296	3.030	0.118	2.078	0.039
	AGI	25.501	2.833	0.534	9.003	<0.001
	CBSMS	−0.122	0.192	−0.037	−0.636	0.526
	FMSS	0.581	0.209	0.144	2.788	0.006
	*R^2^*	0.378				
	Adjusted *R^2^*	0.362				
	ΔR^2^ (*p* < 0.021)	0.019				

CBS, Continuing Bonds Scale (30); AGI, Adolescent Grief Inventory (31); CBSMS, Continuing Bonds on Social Media Scale (1); FMSS, Facebook Measure of Social Support (32); DASS-21, Depression Anxiety Stress Scale 21 (33).

### 3.4 The association between grief-related social media use, perceived social media support, and continuing bonds

Age, sex, type of relationship, emotional closeness, time since death, cause of death, level of grief (AGI), and mental distress (DASS-21) were included in Step 1 [*F* (7,262) = 32.899, *p* < 0.001, *R*^2^ = 0.468]. In Step 2, the use of social media to sustain continuing bonds (CBSMS), and the perceived social support received through social media (FMSS), were included as potential predictors of continuing bonds (CBS) as dependent variable (Table 5). The regression model including step 2 with continuing bonds (CBS) as dependent variable was statistically significant and explained 48.6% of the variance [*F* (9,260) = 27.360, *p* < 0.001, *R*^2^ = 0.486; ΔR^2^ = 0.019, *F* (2, 260) = 4.711, *p* < 0.001]. The use of social media to sustain continuing bonds (CBSMS) (β = 0.165, *p* < 0.001) significantly predicted continuing bonds (CBS). Higher levels of grief-related media use were associated with higher levels of continuing bonds. The perceived social support from social media (FMSS) (β = −0.028, *p* > 0.05) was not a significant predictor of continuing bonds (CBS).

**Table 5:** Hierarchical regression analysis predicting continuing Bonds (CBS) from grief-related social media use and perceived social support on social media (*N* = 270).

Model		Unstandardized coefficients		Standardized coefficients beta	*t*	*p*
		B	Std. Error			
1	(Constant)	−0.350	0.301		−1.164	0.246
	Gender	0.074	0.057	0.060	1.300	0.195
	Age	−0.016	0.012	−0.064	−1.335	0.183
	Relationship to the deceased	−0.027	0.009	−0.139	−2.984	0.003
	Emotional closeness to the deceased	0.139	0.014	0.478	10.104	<0.001
	Months since death	−0.001	0.001	−0.094	−1.941	0.053
	AGI	0.195	0.052	0.218	3.722	<0.001
	DASS−21	0.003	0.001	0.186	3.293	0.001
	*R^2^*	0.468				
	Adjusted *R^2^*	0.454				
2	(Constant)	−0.233	0.316		−0.737	0.462
	Gender	0.058	0.057	0.047	1.012	0.312
	Age	−0.016	0.011	−0.064	−1.362	0.174
	Relationship to the deceased	−0.031	0.009	−0.164	−3.501	<0.001
	Emotional closeness to the deceased	0.129	0.014	0.446	9.264	<0.001
	Months since death	−0.002	0.001	−0.106	−2.211	0.028
	AGI	0.138	0.055	0.154	2.497	0.013
	DASS−21	0.003	0.001	0.185	3.285	0.001
	CBSMS	0.010	0.003	0.165	3.050	0.003
	FMSS	−0.002	0.004	−0.028	−0.574	0.567
	*R^2^*	0.486				
	Adjusted *R^2^*	0.469				
	ΔR^2^ (*p* < 0.010)	0.019				

CBS, Continuing Bonds Scale (30); AGI, Adolescent Grief Inventory (31); CBSMS, Continuing Bonds on Social Media Scale (1); FMSS, Facebook Measure of Social Support (32); DASS-21, Depression Anxiety Stress Scale 21 (33).

## 4 Discussion

This study investigated the associations of grief-related social media use, and perceived social media support, with the grieving process, mental distress, and continuing bonds among bereaved students.

There was a positive correlation between grief-related social media use and perceived social media support. This finding is consistent with the literature. Irwin (26), Rossetto et al. (27), and Stein et al. (22) reported that grief-related social media use can facilitate the receipt of social support from members of online communities. In the study by Rossetto et al. (27), participants identified community building as one of the three primary functions of grief-related Facebook use. Obtaining social support was one of the subcategories of this function (27). Similarly, obtaining social support was identified as a key function of online memorial activities among young adults (22). Bereaved students may also deliberately seek social support on social media by directing their posts to other Facebook users (22).

Regarding the first research question, the correlation analysis revealed a positive association between grief-related social media use and grief reactions. The regression analysis further showed that both grief-related social media use and perceived social media support were significant predictors of grief reactions after controlling for multiple variables including continuing bonds and mental distress. In other words, greater use of social media for grief-related purposes was associated with stronger grief reactions. Conversely, higher levels of perceived social media support predicted less intense grief reactions. These findings are largely consistent with those of Lipp and O'Brien (1). They also found a positive association between grief-related social media use and the intensity of grief reactions, and identified grief-related social media use, but not perceived social media support, as a significant predictor of grief reactions (1).

Our findings differ from what other authors have called the “coping paradox” (27). Facebook and other social media allow for the expression of grief and sharing stories, which may facilitate the grief process (26). However, bereaved individuals can be at risk of maladaptive coping when they struggle to differentiate the new virtual continuing bond from the past relationship and engage in rumination because of the ongoing online presence and continuing communications (1, 27).

Regarding the second research question, our study found that both grief-related social media use and perceived social media support were positively correlated with levels of mental distress. This finding is supported by the studies of Rossetto et al. (27), Stein et al. (22), and Visser and Parrott (37). Furthermore, perceived social media support was found to be a significant predictor of lower levels of mental distress. This finding was different from the findings of the study by Lipp and O'Brien (1), in which perceived social media support was not found to be a predictor of posttraumatic growth, a factor that may contribute to enhanced mental health. Our findings suggest that those with the highest levels of distress may be more likely to use social media for support. Other studies reported that a family history of mental illness may also predict such help-seeking (38). Nonetheless, the overall evidence of the effect of social media use on mental health in young people is still equivocal (39). Further research, for example through longitudinal studies might be needed to elucidate the association between perceived social media support and mental health in bereaved students.

Regarding the third research question, our study found a positive correlation between grief-related social media use and continuing bonds. This finding is partly corroborated by Lipp and O'Brien (1), who reported a significant positive correlation between maintaining continuing bonds on social media and perceived and emotional social media support, while we did not find a correlation between perceived social media support and continuing bonds. The studies by Irwin (26), Rossetto et al. (27), and Stein et al. (22) also point to a positive association between grief-related social media use and continuing bonds. Social media would enable individuals to maintain continuing bonds with the deceased regardless of time and place (26, 27).

Irwin (26) further found that young adults actively use social media to maintain continuing bonds on social media because they believe in the possibility of an ongoing connection with the deceased. Similarly, Rossetto et al. (27) and Stein et al. (22) identified continuing bonds as one of the primary functions of grief-related social media use, with participants intentionally directing their messages to the deceased to sustain a continuing bond (22, 27).

### 4.1 Implications

Grief-related social media use is positively associated with the intensity of grief reactions and levels of mental distress. In addition, higher levels of grief-related social media use were found to predict stronger grief reactions. These findings indicate the importance of professional and peer supporters paying close attention to the social media use of bereaved students.

This study also found a positive correlation between grief-related social media use and perceived social media support. Still, support providers should consider the potential negative impact of grief-related social media use as well, because obtaining social support through social media was positively associated with, and a predictor of poorer mental health.

Perceived social media support was found to be a negative predictor of grief reactions. Professional and peer supporters should therefore consider that grief-related social media use and perceived social media support may not necessarily have positive consequences. Extensive grief-related social media use may be associated with difficulties adapting to the loss and increase the risk of prolonging grief-related distress and development or exacerbation of mental health problems (1, 40).

The nature of social support itself is also important. Students may experience support on social media negatively. For example, when peers address the topic of bereavement too intensely or too frequently, bereaved students may repeatedly re-live the pain associated with the loss. Thus, professional and peer supporters might consider both grief-related social media use and perceived social media support among bereaved students. This may enable support providers to detect and respond to distress and potentially adverse grief reactions at an early stage.

Furthermore, this study demonstrated that grief-related social media use was positively correlated with and predicted continuing bonds. Given that contemporary grief models emphasize the importance of continuing bonds (18, 41), it may be beneficial to investigate how digital ways of maintaining these bonds can be incorporated into interventions and support programs. By learning students how to engage with social media in a constructive manner to maintain continuing bonds with the deceased, the sense of an ongoing connection may be fostered and strengthened.

### 4.2 Limitations

This study contributes to the scarce literature on grief-related social media use in students. A few limitations should be considered. Although one in five participants was male, the sample was predominantly female and relied on voluntary participants, which may limit the generalizability of the findings. Those who felt more comfortable in discussing their grief experiences and using social media may have been more likely to participate than others. The survey included several retrospective self-report questionnaires, which may have introduced recall bias, and psychometric validation of the translated measures was not conducted. The cross-sectional design does not allow us to draw conclusions about the direction of the associations. Future longitudinal research could offer further insights into how relationships between grief-related social media use, perceived social media support, the grieving process, mental distress, and continuing bonds develop over time among bereaved students.

## 5 Conclusions

Grief-related social media use positively predicted stronger grief reactions and stronger continuing bonds with the deceased among bereaved students. Perceived social media support predicted less severe grief reactions, but higher levels of mental distress. The findings indicate that bereaved students who engage more in grief-related social media activities may be at increased risk of experiencing grief and mental health problems. By exploring the specific needs and coping strategies of bereaved students who engage with or seek support through social media, practitioners may play a crucial role in contributing to healthy coping and positive mental health outcomes.

## Data Availability

The datasets presented in this article are not readily available because researchers are not allowed to share the data beyond the research team. Requests to access the datasets should be directed to karl.andriessen@unimelb.edu.au.
